# Statins Reduce Lipopolysaccharide-Induced Cytokine and Inflammatory Mediator Release in an In Vitro Model of Microglial-Like Cells

**DOI:** 10.1155/2017/2582745

**Published:** 2017-05-04

**Authors:** A. J. McFarland, A. K. Davey, S. Anoopkumar-Dukie

**Affiliations:** ^1^Menzies Health Institute Queensland, Griffith University, Gold Coast, QLD, Australia; ^2^School of Pharmacy, Griffith University, Gold Coast, QLD, Australia; ^3^Quality Use of Medicines Network, Gold Coast, QLD 4222, Australia

## Abstract

The anti-inflammatory effects of statins (HMG-CoA reductase inhibitors) within the cardiovascular system are well-established; however, their neuroinflammatory potential is unclear. It is currently unknown whether statins' neurological effects are lipid-dependent or due to pleiotropic mechanisms. Therefore, the assumption that all statin compounds will have the same effect within the central nervous system is potentially inappropriate, with no studies to date having compared all statins in a single model. Thus, the aim of this study was to compare the effects of the six statins (atorvastatin, fluvastatin, pitavastatin, pravastatin, rosuvastatin, and simvastatin) within a single in vitro model of neuroinflammation. To achieve this, PMA-differentiated THP-1 cells were used as surrogate microglial cells, and LPS was used to induce inflammatory conditions. Here, we show that pretreatment with all statins was able to significantly reduce LPS-induced interleukin (IL)-1*β* and tumour necrosis factor (TNF)-*α* release, as well as decrease LPS-induced prostaglandin E2 (PGE2). Similarly, global reactive oxygen species (ROS) and nitric oxide (NO) production were decreased following pretreatment with all statins. Based on these findings, it is suggested that more complex cellular models should be considered to further compare individual statin compounds, including translation into in vivo models of acute and/or chronic neuroinflammation.

## 1. Introduction

Statins, or HMG-CoA reductase inhibitors, are widely used agents in the treatment of dyslipidaemia and the prevention of cardiovascular disease (CVD). It is well-established that this class of medications has a broad and potent effect on the lipid profile, as well as the ability to halt atherosclerotic disease progression; both of which contribute to reduced CVD risk in patients [1]. Additionally, recent evidence suggests that it is the cholesterol-independent, or “pleiotropic,” effects of statins which account for many of their cardioprotective properties.

A number of pleiotropic effects have been documented across in vitro, in vivo, and clinical studies thus far. These include improved endothelial function and favourable effects on vascular redox state [2, 3], stabilisation of atherosclerotic plaques [4], and inhibition of the thrombogenic response [5, 6]. However, it is statins' ability to reduce oxidative stress and inflammation which is believed to contribute to the majority of their pleiotropic benefits both within and beyond the cardiovascular system [7, 8]. Independent of lipid-level changes, statins have been clinically associated with a reduction in plasma C-reactive protein levels and decreased circulating proinflammatory cytokines interleukin (IL)-1, IL-6, and tumour necrosis factor (TNF)-*α* [9–11] in patients with chronic disease. Furthermore, in vitro and in vivo studies have identified that statins were able to reduce cyclooxygenase (COX)-2 and matrix metalloproteinase (MMP)-9 activity [12, 13], improve nitric oxide (NO) bioavailability [2], reduce nuclear factor-*κ*B (NF-*κ*B) activation [14], and upregulate cellular antioxidant enzymes [15, 16] in various models of induced inflammation. Clinically, the anti-inflammatory properties of statins have been implicated as a central mechanism for statins' effects in primary and secondary stroke prevention [17, 18], cardioprotection in coronary artery disease [19], and improvements of short-term outcomes in acute coronary syndrome [20].

Whilst the effects of statins within the cardiovascular system are well-established, their effects on other body systems remain less clear. Within the central nervous system (CNS), a number of conflicting neurological outcomes have been reported following statin use, including acute memory loss [21], protection against long-term cognitive decline [22], cognitive improvements following cerebral malaria [8], and improvements in multiple sclerosis [23]. Whilst the role of statins in inducing memory loss has now been largely refuted [24], statins' anti-inflammatory effects have been suggested as a possible mechanism for statin-induced neuroprotection seen in many studies [7]. Both in vitro and in vivo studies have shown individual statins to be associated with a range of anti-inflammatory mechanisms across various experimental neuroinflammation models [7]; these include reduced microglial activation and reduced release of inflammatory mediators such as TNF-*α*, IL-1*β*, and NO [25–31]. Whilst studies to date provide some insight as to the antineuroinflammatory potential of statins, it is currently unclear whether these effects are lipid-dependent or due to pleiotropic mechanisms. As such, results from existing models where select statin(s) have been used may not be representative of all statins as a class, particularly given the structural and chemical diversity of these compounds [7], as briefly outlined in Table 1. In addition to structural differences, these compounds differ in their respective half-lives, volumes of distribution, tendencies for protein binding, and metabolism pathways [7]. Furthermore, our current understanding of the antineuroinflammatory potential of these compounds is limited by differences in the experimental model used in available studies, including use of different cell lines, neuroinflammatory inducers, durations of exposure, and/or outcome measure(s). For example, whilst rosuvastatin treatment has been shown to reduce the production of neuroinflammatory cytokines in cultured microglia [26], simvastatin was shown to decrease IL-1*β* but not TNF-*α* in a similar primary rat microglial model, where a lower dose of lipopolysaccharide (LPS) was used for a shorter time period [32]. As such, it is difficult to accurately compare the neuroinflammatory potential of statins given the currently available evidence.

Whilst statins do appear to have some anti-inflammatory effects in the CNS based on the currently available literature, our knowledge of how these individual compounds compare is limited. Thus, the aim of this study was to compare the effects of multiple statin compounds within a single in vitro model of neuroinflammation. To achieve this, we used phorbol 12-myristate 13-acetate- (PMA-) differentiated THP-1 cells as a well-established in vitro surrogate microglial model and determined the effects of statins on LPS-induced inflammation of this model. To determine whether any observed effects were compound-specific or applicable to the whole class, we used a panel of structurally and pharmacologically diverse statins, including atorvastatin, fluvastatin, pitavastatin, pravastatin, rosuvastatin, and simvastatin.

## 2. Methods

### 2.1. Cell Culture and Differentiation

Human peripheral blood monocytes from acute monocytic leukaemia cells (THP-1) were obtained from Sigma (St. Louis, MI, USA). Cells were cultured and maintained at 37°C in 5% CO_2_ in complete Roswell Park Memorial Institute (RPMI) 1640 media containing glucose, sodium pyruvate, L-glutamine, (4-(2-hydroxyethyl)-1-piperazineethanesulfonic acid (HEPES) buffer, and Phenol Red (Invitrogen, Victoria, Australia), supplemented with 10% foetal bovine serum (Invitrogen, Victoria, Australia), 0.05 mM 2-mercaptoethanol (Sigma, St Louis MO, USA), and 0.5 mg/L gentamicin (Invitrogen, Victoria, Australia). Cells were subcultured to maintain a cell suspension density of between 2.0 × 10^5^ and 1.0 × 10^6^ cells/mL. All experiments were conducted using this medium except 2′,7′-dichlorofluorescein diacetate (DCFH-DA) fluorometry. Differentiation of THP-1 cells was optimised based on previously described protocols using PMA (also termed 12-O-tetradecanoylphorbol-13-acetate or TPA) [33–36]. Briefly, cells were seeded at 3.0 × 10^5^ trypan blue-excluding cells/mL as per individual experiment requirements. At 24 h, cells were treated with PMA (10–200 nM) and left for 5 days, with a media change at 72 h after initial seeding.

### 2.2. Statin Treatments and LPS-Induced Neuroinflammation

To induce conditions of neuroinflammation, LPS from *Escherichia coli*, serotype 055:B5 (Sigma, St. Louis, MI, USA), was used. LPS is a potent immune stimulant and has been widely used in models of microglial activation and neuroinflammation [37–40]. Following differentiation, cells were treated with either vehicle control or one of the six statins (atorvastatin, fluvastatin, pitavastatin, pravastatin, rosuvastatin, or simvastatin; 0–100 *μ*M) and incubated at 37°C in 5% CO_2_ for 60 minutes. After this time, cells were treated with LPS (0–100 *μ*g/mL) and incubated at 37°C in 5% CO_2_ for 24 h.

### 2.3. FITC-LPS Fluorometry

To determine whether statin administration was interfering with LPS binding to the differentiated THP-1 cells, fluorescein isothiocyanate- (FITC-) conjugated LPS from *E. coli*, serotype 055:B5 (Sigma, St. Louis, MI, USA), was used as previously described [41]. Briefly, cells were treated with either vehicle control or one of the six statins (atorvastatin, fluvastatin, pitavastatin, pravastatin, rosuvastatin, or simvastatin; 0–100 *μ*M) following PMA differentiation and incubated at 37°C in 5% CO_2_ for 60 minutes. After this time, cells were treated with FITC-labelled LPS (0–100 *μ*g/mL) and incubated at 37°C in 5% CO_2_ for 30 minutes. Following incubation, cells were washed and with PBS, and fluorescence intensity quantified using a Fluoroskan Ascent microplate fluorometer (excitation: 485 nm, emission: 590 nm) (Thermo Scientific, Victoria, Australia).

### 2.4. Resazurin (Alamar Blue) Proliferation Assay

Resazurin is a nonfluorescent compound which is reduced to resorufin, a fluorescent dye, in the presence of metabolically active cells and thus can be used as a measure of cellular proliferation [42]. Following LPS treatment as described previously, media above cells were replaced with 200 *μ*L complete media containing 44 *μ*M resazurin, and plates incubated at 37 °C, 5% CO_2_ were protected from light for 4 h. After this incubation, reduction of resazurin to resorufin was quantified using a Fluoroskan Ascent microplate fluorometer (excitation: 530 nm, emission: 590 nm) (Thermo Scientific, Victoria, Australia). Appropriate cell-free controls were included. Results from each tested condition were validated using a Countess® automated cell counter (Invitrogen, Victoria, Australia), with the extent of resazurin reduction proportional to viable cell counts (data not shown).

### 2.5. Determination of IL-1*β*, PGE2, and TNF-*α*

THP-1 cells were differentiated and treated with statins and LPS as previously described. Following 24 h LPS treatment, IL-1*β* and TNF-*α* were measured in 100 *μ*L of cell supernatant by enzyme immunoassay (EIA; Cayman Chemical Company, Ann Arbor, MI, USA). PGE2 was measured in 50 *μ*L of cell supernatant by express EIA (Cayman Chemical Company, Ann Arbor, MI, USA). All steps were performed as per manufacturer's instructions. Absorbance was measured using a Tecan Infinite 200 Pro microplate reader (Tecan, Victoria, Australia). All reported values were above the limit of detection range specified by the manufacturer. Samples were diluted in culture media as per manufacturer's instructions to ensure that results obtained were within the limit of quantification. Appropriate controls were used to determine possible interference from individual statins and/or LPS with the absorbance reading.

### 2.6. DCFH-DA Fluorometry

2′,7′-Dichlorofluorescein diacetate (DCFH-DA) was used to measure global ROS production as previously described [43]. Following 24 h LPS treatment as previously described, the culture media above cells was replaced with serum-free media containing DCFH-DA (10 *μ*M/L) for 30 minutes. Cells were then washed twice using phosphate-buffered saline (PBS) and fluorescence (excitation: 485 nm, emission: 535 nm) measured using a Fluoroskan Ascent microplate fluorometer (Thermo Scientific, Victoria, Australia).

### 2.7. Nitric Oxide Production

Nitric oxide production was measured fluorometrically using 4,5-diaminofluorescein diacetate (DAF-2 DA). DAF-2 DA is a cell permeable nitric oxide probe which is hydrolysed to fluorescent 4,5-diaminofluorescein (DAF-2) in the presence of nitric oxide. THP-1 cells were differentiated and treated with statins and LPS as previously described. Following 24 h LPS treatment, culture media above was replaced with serum-free media containing 10 *μ*M DAF-2 DA (Cayman Chemical Company, Ann Arbor, MI, USA) and incubated in the dark for 45 minutes (37°C, 5% CO_2_). Following incubation, cells were washed twice with PBS and fluorescence (excitation: 485 nm, emission: 538 nm) measured using a Tecan Infinite 200 Pro microplate reader (Tecan, Victoria, Australia).

### 2.8. Statistical Analyses

Data were analyzed by one-way analysis of variance (ANOVA) with the Tukey-Kramer multiple comparisons test, using GraphPad InStat software v3.06 (San Diego, California). Significance levels were defined as *P* < 0.05 (^∗^), *P* < 0.01 (^∗∗^), and *P* < 0.001 (^∗∗∗^). All graphs were drawn using GraphPad Prism v6.01 (San Diego, California).

## 3. Results

### 3.1. PMA-Differentiated THP-1 Cells Behave in a Microglial-Like Manner in an LPS Model of Neuroinflammation

The use of PMA-differentiated THP-1 human monocyte cells as surrogate microglia is well-recognised and accepted, given that primary human microglia are difficult to obtain in large quantities [44–46]. Differentiation of THP-1 cells to an activated, microglial-like cell was achieved through use of the phorbol ester PMA. Consistent with microglia, differentiated THP-1 (dTHP-1) cells became adherent and exhibited concentration-dependent phenotypic changes, including round morphology and increasing diameter with increasing PMA concentrations (data not shown). All subsequent experiments used 100 nM PMA to differentiate THP-1 cells.

**Figure 1:**
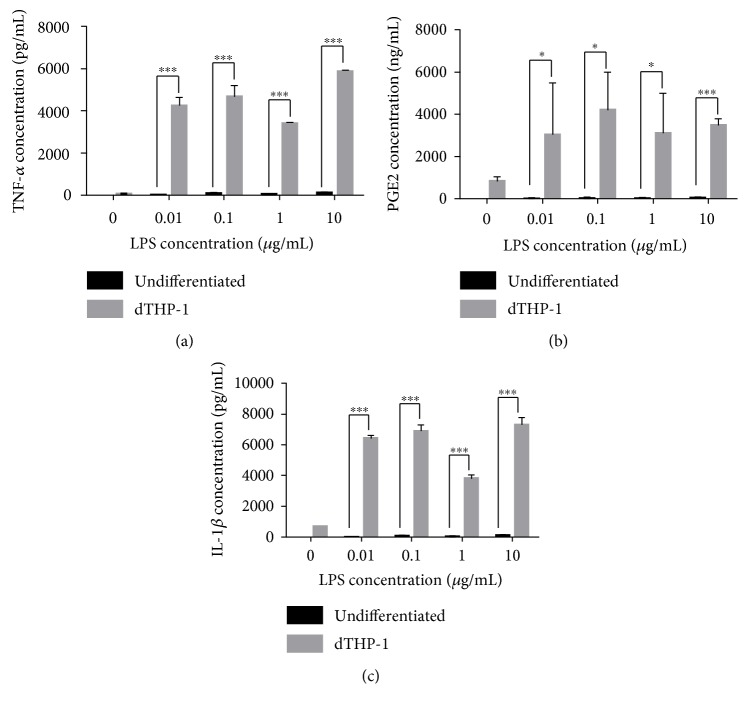
Verification of proinflammatory mediator release following LPS activation of differentiated THP-1 (dTHP-1) cells. Cells were treated with LPS (0–10 *μ*g/mL) for 24 h, following the release of (a) TNF-*α*, (b) PGE2, and (c) IL-1*β* which were measured by ELISA. Data shows mean + SD of three independent experiments.

Exposure of dTHP-1 to LPS from *E. coli* 055:B5 (0.01–10 *μ*g/mL) was used to replicate well-established in vitro neuroinflammation models [47, 48]. As seen in Figure 1, LPS exposure (0.01–10 *μ*g/mL) produced significant release of IL-1*β*, TNF-*α*, and PGE2 (*P* < 0.05). An LPS concentration of 0.1 *μ*g/mL was used in all subsequent experiments to induce neuroinflammatory conditions. LPS did not induce any changes in cellular proliferation as measured through the reduction of resazurin to resorufin (data not shown).

### 3.2. Statins Attenuate LPS-Induced TNF-*α*, IL-1*β*, and PGE2 Release in Microglial-Like dTHP-1 Cells

FITC-labelled LPS was used to determine whether statins interfered with the binding of LPS to THP-1 cells in our model. There was no statistically significant change in fluorescence in FITC-LPS-only treated cells compared to cells which were pretreated with statins (supplementary figure available online at https://doi.org/10.1155/2017/2582745). Statin-induced changes in dTHP-1 cellular proliferation were measured through the use of the resazurin reduction assay, and all subsequent results were normalised to cell number. To determine the individual effects of statins on LPS-induced inflammatory mediator production, dTHP-1 cells were pretreated with one of the six statins prior to LPS treatment, and subsequent IL-1*β*, TNF-*α*, and PGE2 release were measured after 24 hours. Control experiments found that neither the statin compounds nor LPS caused interference with absorbance readings from any of the three assays (data not shown).

**Figure 2:**
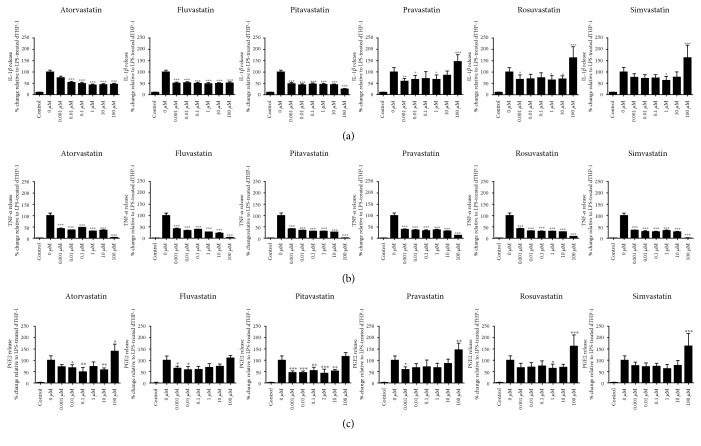
Statin-induced changes in (a) IL-1*β*, (b) TNF-*α*, and (c) PGE2 release in LPS-activated dTHP-1 cells. Cells were pretreated for 24 h with one of the six statins (0–100 *μ*M) prior to receiving a 24 h LPS stimuli, after which time IL-1*β*, TNF-*α*, and PGE2 release were quantified by ELISA. Control cells represent cells treated with statin and LPS vehicles. Data shown is mean + SD of four independent experiments; vehicle control (0 *μ*M) is represented as mean + SEM. ^∗^Statin treatment versus LPS only.

As shown in Figure 2(a), atorvastatin, fluvastatin, and pitavastatin significantly reduced IL-1*β* release relative to LPS-only treated cells at all tested concentrations. Whilst pravastatin, rosuvastatin, and simvastatin were shown to reduce IL-1*β*; it was, to a lesser extent, with not all changes found to be significant. Additionally, at 100 *μ*M, the highest tested concentration, pravastatin, rosuvastatin, and simvastatin were shown to increase IL-1*β* release (Figure 2(a); *P* < 0.001), though this was not significant. All statins at all concentrations were shown to significantly decrease TNF-*α* release (*P* < 0.001) relative to the LPS-treated control (Figure 2(b)). Similarly, all statins were able to attenuate LPS-induced PGE2 release, though not at all measured concentrations (Figure 2(c)). In contrast, the highest tested concentration (100 *μ*M) of atorvastatin, pravastatin, rosuvastatin, and simvastatin significantly increased PGE2 release from LPS-stimulated dTHP-1 cells (*P* < 0.05).

### 3.3. Statins Reduce Global Reactive Oxygen Species and Nitric Oxide Production

**Figure 3:**
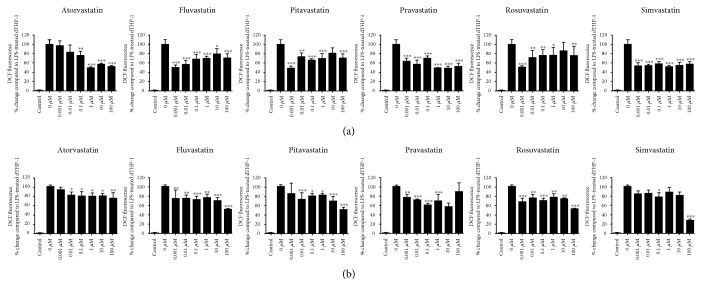
Statin-induced changes in (a) DCF fluorescence, as a measure of global ROS production, and (b) DAF fluorescence, as a measure of nitric oxide release, in LPS-activated dTHP-1 cells. Cells were pretreated for 24 h with one of the six statins (0–100 *μ*M) prior to receiving a 24 h LPS stimuli, after which time DAF and DCF fluorescence were measured. Statin-treated data is represented as mean + SD; vehicle control (0 *μ*M) is represented as mean + SEM. All values represent the results from three independent experiments. ^∗^Statin treatment versus LPS only.

In addition to the production of proinflammatory cytokines and prostaglandin E_2_, microglia are also known to produce various nitrosative and oxidative species as part of the neuroinflammatory response. Thus, global ROS and nitric oxide production were screened alongside changes in cytokines and PGE2. Following LPS treatment, the production of global ROS in dTHP-1 cells as quantified through DCF fluorescence was significantly increased compared to that in the untreated dTHP-1 controls (Figure 3(a)). All statins were shown to significantly attenuate LPS-induced DCF fluorescence (Figure 3(a)); however, only fluvastatin, pravastatin, and simvastatin were found to significantly decrease DCF fluorescence at all concentrations. To determine the effects on NO release, DAF-2 fluorescence was used. LPS treatment in the dTHP-1 cells significantly increased DAF-2 fluorescence, indicative of increased NO release. All statins were shown to decrease DAF-2 fluorescence to some extent (Figure 3(b)). At the highest concentrations of fluvastatin, pitavastatin, rosuvastatin, and simvastatin, up to 50% reduction in DAF fluorescence was observed, suggesting a dose-dependent response. Conversely, atorvastatin treatment decreased DAF-2 fluorescence to approximately 80% across all concentrations, with increases in dose eliciting no apparent dose-dependent effects. Control experiments found that neither the statin compounds nor LPS caused interference with fluorescence readings from the DCFH-DA and DAF-2-DA assays (data not shown).

## 4. Discussion

Statins are amongst the most widely prescribed medications worldwide, indicated both in dyslipidaemia and in a number of non-lipid-related cardiovascular conditions, such as high CVD risk and postmyocardial infarction [49–52]. Recently, several lines of evidence have additionally suggested that statins may also have a role in the treatment or prevention of certain neurological disorders, including multiple sclerosis and Alzheimer's disease, due to their anti-inflammatory effects [8, 23]. Evidence for statins' anti-inflammatory effects within CNS models are conflicting, and inconsistencies between findings attributed to several factors, including the potential for effect variability between individual statin compounds [7]. The extent of this limitation is unclear, as, to our knowledge, no studies to date have compared the anti-inflammatory potential of multiple statins in a single model. Therefore, the aim of this study was to compare the effects of the six individual statins (atorvastatin, fluvastatin, pitavastatin, pravastatin, rosuvastatin, and simvastatin) in an in vitro microglial-like model of LPS-induced neuroinflammation.

Our results demonstrate that all six statins exhibit similar anti-inflammatory potential, reducing cytokines IL-1*β* and TNF-*α*, as well as PGE2. ROS production and NO release were similarly reduced by all six statins in the present model. As statin treatment did not affect FITC-labelled LPS fluorescence, the reduction in antineuroinflammatory mediators can be attributed to an anti-inflammatory response as opposed to a blockage in LPS-induced effects. Neuroinflammation is considered to play a central role in the pathogenesis of a number of neurological disorders, including depression, neurodegenerative disorders, multiple sclerosis, and neuropathies [53–55]. The elevations of both IL-1*β* and TNF-*α* are recognised as critical components in the neuroinflammatory response, with both mediators shown to independently induce neuronal cell death [56, 57]. Hence, statins' ability to reduce both of these factors to a similar extent in our model has several implications for neuroinflammation and its pathogenesis. IL-1*β* is considered to be a major proinflammatory cytokine within the brain and is a key regulator of acute inflammatory processes [58, 59]. Whilst neurons, astrocytes, endothelial cells, and oligodendrocytes may also be capable of producing IL-1*β*, this production appears secondary to release from microglia; hence, inhibition at the microglial stage may reduce amplification responses [59]. Similar to IL-1*β*, the de novo release of TNF-*α* from activated microglia induces a cascade of events which perpetuate the inflammatory response. Whilst astrocytes and neurons are both able to produce TNF-*α*, it is widely acknowledged that microglia are the predominant source during neuroinflammation [60, 61]. Despite TNF-*α* being reduced at all tested concentrations of statins, IL-1*β* release was augmented by pravastatin, rosuvastatin, and simvastatin (100 *μ*M). Statin-induced augmentation of IL-1*β* has been described previously in other models, with pravastatin increasing IL-1*β* in a murine model of lung injury and fibrosis [25]. This may suggest a dose-dependent proinflammatory effect, and this finding warrants further investigation. Overall, the findings of this study are consistent with previous studies which have found rosuvastatin and simvastatin to inhibit TNF-*α* and IL-1*β* release from microglia [32, 26]; however, here, we show that atorvastatin, fluvastatin, pitavastatin, and pravastatin exert a similar effect on our model, suggesting that these compounds also be of potential therapeutic benefit in attenuating neuroinflammatory disease.

Unlike cytokines IL-1*β* and TNF-*α*, PGE2 has a paradoxical role in neuroinflammation, with evidence for its contribution to both the progression and the resolution of inflammation in the neuroparenchyma [62–64]. Within the CNS, the synthesis of PGE2 can assist in mediation of bradykinin-induced neuroprotection, with a documented ability to reduce cytokine release from cultured glial cells and microglia [65, 66]. As such, PGE2 has an important role in the resolution of acute injury; however, chronically elevated levels of this eicosanoid can result in neuronal injury, resulting in lesions and enhancing pain transmission [67, 68]. In the present study, we find that all six statins are able to decrease PGE2 at lower concentrations; however, at the maximal concentration (100 *μ*M), PGE2 release was either unaffected or elevated beyond LPS induction alone. Statin-induced increases in PGE2 production have been reported in nonneurological models, although these have been in models of acute in vivo inflammation via experimental rotator cuff injury [69]. Again, further investigation may be warranted to clarify whether the observations from the present study are due to dose-dependent effects. Additionally, the therapeutic relevance of this concentration range of statins within the CNS is unknown; with plasma concentrations of statins known to range from 1 to 15 nM/L [70].

Similar to PGE2, we see a dual role for ROS and reactive nitrogen species (RNS) in the neuroparenchyma. At low concentrations, both ROS and RNS are essential regulatory mediators in signal processing; the excess production of these mediators, however, is associated with a plethora of neuroinflammatory and neurodegenerative diseases [71, 72]. ROS act as critical-signalling molecules in the initiation of neuroinflammatory responses through activation of redox-sensitive transcription factors such as NF-*κ*B and activator protein-1 (AP-1), which further propagates the inflammatory response [72–74]. Our data suggest that all six statins tested have the potential to reduce ROS and NO production, although the extent and dose-dependent nature of the reduction varies between statin compounds. This finding is consistent with a number of studies where statins are implicated as having an antioxidant and NO-reducing effects. Simvastatin has previously been shown to reduce LPS-induced NO, inducible nitric oxide synthase (iNOS), and ROS production in BV-2 microglial cells [27] and decrease endothelial nitric oxide synthase (eNOS) in murine foecal cerebral ischaemia [75]. In addition, atorvastatin was found to significantly reduce lipoperoxidation and protein nitration in the parietal cortex in vivo [28]. Despite the currently available evidence, our study is the first to our knowledge which compares each statin compound individually in a single model of neuroinflammation.

## 5. Conclusion

Given the findings of the present study, statins' abilities to reduce production of a range of known proinflammatory mediators in a surrogate microglial model have a number of potential clinical implications. All six statins tested were found to exhibit similar anti-inflammatory and antioxidant activity, suggesting that this class may have similar activity against the consequences of induced microglial activation. As neuroinflammation is a multicellular process, it is necessary to consider that whilst the present study suggests that all of the six statins tested may have similar anti-inflammatory effects on microglial-like cells in vitro, statins' effects on other cell types, and subsequent cell-cell interactions, have yet to be considered. Based on the results and limitations of the present study, it is suggested that more complex cellular models should be considered, including translation into in vivo models of acute and/or chronic neuroinflammation.

## Supplementary Material

Supplementary Figure 1. Treatment of differentiated THP-1 cells with FITC-conjugated LPS following statin pre-treatment. Cells were treated with 0.1 μg/mL LPS for 30 minutes, following which cells were washed and fluorescence determined. Data shows mean + SD of three independent experiments.

## Figures and Tables

**Table 1:** Chemical structure and properties of the commonly prescribed statins [7].

Statin	Molecular structure	Lipophilicity	Half-life
Atorvastatin	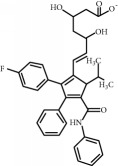	Lipophilic	14 h
Fluvastatin	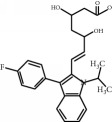	Lipophilic	2.3 h
Pitavastatin	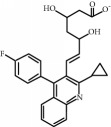	Lipophilic	12 h
Pravastatin	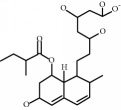	Hydrophilic	2.7 h
Rosuvastatin	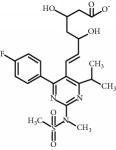	Hydrophilic	19 h
Simvastatin	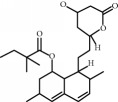	Lipophilic	3 h
