# Supplementary material for: Statins Reduce Lipopolysaccharide-Induced Cytokine and Inflammatory Mediator Release in an In Vitro Model of Microglial-Like Cells
Source: Mediators Inflamm. 2017 May 4;2017:2582745. doi: 10.1155/2017/2582745 (PMC5435995; doi:10.1155/2017/2582745)
Supplement: Supplementary file 1 — Supplementary Figure 1. Treatment of differentiated THP-1 cells with FITC-conjugated LPS following statin pre-treatment. Cells were treated with 0.1 μg/mL LPS for 30 minutes, following which cells were washed and fluorescence determined. Data shows mean + SD of three independent experiments. [file 2582745.f1.docx]

**Supplementary Figure 1:** Treatment of differentiated THP-1 cells with FITC-conjugated LPS following statin pre-treatment. Cells were treated with 0.1 µg/mL LPS for 30 minutes, following which cells were washed and fluorescence determined. Data shows mean + SD of three independent experiments.
